# Severity and Risk Factors Associated with Premature Coronary Artery Disease in Patients Under the Age of 50 in Saudi Population: A Retrospective Study

**DOI:** 10.3390/jcm14051618

**Published:** 2025-02-27

**Authors:** Thamir Al-khlaiwi, Syed Shahid Habib, Hessah Alshammari, Hanan Albackr, Razan Alobaid, Lama Alrumaih, Faye Sendi, Shahad Almuqbil, Muhammad Iqbal

**Affiliations:** 1 Department of Physiology, College of Medicine, King Saud University, Riyadh 11461, Saudi Arabia; sshahid@ksu.edu.sa (S.S.H.); 441200337@student.ksu.edu.sa (R.A.); 441200593@student.ksu.edu.sa (L.A.); 441200588@student.ksu.edu.sa (F.S.); 441200388@student.ksu.edu.sa (S.A.); 2 Department of Cardiology Science, College of Medicine, King Saud University, Riyadh 11461, Saudi Arabia; healshammari@ksu.edu.sa; 3 Department of Cardiac Sciences, King Fahad Cardiac Center, College of Medicine, King Saud University Medical City, King Saud University, Riyadh 19910, Saudi Arabia; halbackr@ksu.edu.sa

**Keywords:** premature coronary artery disease, age, smoking, hyperlipidemia

## Abstract

**Background and Objectives**: The average age of presentation of coronary artery disease (CAD) is one decade younger in the Saudi population relative to other patients worldwide. It is imperative to investigate the prevalence of premature coronary artery disease (PCAD) risk factors in Saudi Arabia’s younger population in order to prevent the incidence of cardiovascular diseases in the future. Thus, the present study aimed to evaluate the severity and identify the risk factors associated with PCAD in patients under the age of 50 at King Saud University Medical City (KSUMC), Saudi Arabia. **Methods**: This observational retrospective study was conducted between June 2022 and June 2023 at King Saud University Medical City, Riyadh, Saudi Arabia. A total of 718 participants were included in the study. The patients, confirmed by electrocardiographic and/or angiographic findings of coronary artery disease, were divided into three age groups: group 1 (<40 years), group 2 (40–45 years), and group 3 (45–50 years). The severity of vessel occlusions was evaluated using the Gensini scoring system. Electrocardiographic findings, sociodemographic variables, and risk factors were also taken into consideration. **Results**: The mean age of patients in group 1 was 35.2 ± 4.5 years, in group 2 was 43.0 ± 1.3 years, and in group 3 was 48.4 ± 1.4 years. Patients in group 2 had a significantly higher BMI (31.3 ± 10.5) compared to patients in group 3 (29.4 ± 5.3; *p* = 0.015). Nearly 55% of patients under 40 years had 2 or 3 vessel occlusions according to the vessel score. The percentage of patients with inferior ST elevation was significantly higher in group 1 (<40 years, 11.2%) compared to groups 2 (40–45 years, 10.1%) and 3 (45–50 years, 6.0%; *p* = 0.001). Non-specific ST-T changes were more common in group 1 (31.4%) and group 2 (32.0%) compared to group 3 (28.4%). Although not statistically significant, left main artery occlusion tended to be higher in group 3 (8.6%) compared to groups 1 (4.6%) and 2 (4.5%; *p* = 0.229). Hyperlipidemia levels were significantly higher in patients with a Gensini score > 39 compared to those with a Gensini score < 39 (47.9% vs. 37.5%, respectively; *p* = 0.05). The prevalence of smoking was about 54% in group 1, followed by type 2 diabetes mellitus, dyslipidemia, and hypertension (37%, 36%, and 33%, respectively). **Conclusions**: This study suggested that PCAD Saudi patients below 40 years of age had a higher percentage of inferior ST elevation compared to older patients, while non-specific ST-T changes were significantly higher in older patients. Astonishingly, more than 50% of patients in all groups had two or three vessel occlusions. There was a high prevalence of modifiable risk factors, such as smoking, in younger patients, whereas hyperlipidemia was a risk factor for PCAD in all age groups. In addition, hyperlipidemia was highly correlated with severe vessel occlusion according to the Gensini score. Therefore, early preventive measures should be taken into consideration to reduce the future burden of cardiovascular complications in this population.

## 1. Introduction

Cardiovascular diseases (CVDs) are the main type of non-communicable diseases, which account for more deaths than any other disease globally [1]. CVDs comprise disorders of heart and blood vessels, such as coronary artery disease, congenital heart disease, peripheral artery disease, rheumatic heart disease, and cerebrovascular disease.

Coronary artery disease (CAD) is an important cause of death and loss of disability-adjusted life years (DALYs) around the globe [2,3]. In Saudi Arabia, data are scarce regarding the prevalence of CAD. An earlier study estimated an overall prevalence of 5.5% CAD in individuals aged between 30 and 70 years [4]. However, higher estimates project a 24–30% prevalence of CAD in the Saudi population, depending upon age, risk factors, and study settings [5,6]. Early onset of CAD in young adults, ranging from 45 to 55 years of age, is usually termed as premature coronary artery disease (PCAD) [7,8,9,10].

There is no universally accepted definition of PCAD, as different studies have reported different age limits to describe its occurrence locally and globally, which affects its prevalence and associated risk factors.

The prevalence of PCAD is around 5–10% among CAD patients worldwide [11,12] and, more specifically, 11% in the Middle East, Africa is 9.7%, North America is 4%, and Western Europe is 2.7% [13,14]. Unfortunately, the literature is lacking regarding the prevalence of PCAD in Saudi Arabia even though some studies conducted in Saudi Arabia found that 49% of ST-elevation myocardial infarction (STEMI) occurred under 45 years of age [15], while another study found that 16.7% of patients presenting with acute coronary disease were younger than 45 years [16].

Various modifiable and non-modifiable risk factors are associated with PCAD and CAD. Conventional risk factors, such as smoking, a family history of CAD, opium use, and dyslipidemia, are considered more dominant in PCAD, compared to factors like a sedentary lifestyle, hypertension, obesity, diabetes mellitus, smoking, and a lack of awareness, which are more commonly found in individuals with late-onset disease [17,18].

Young people usually have less information regarding risk factors linked with cardiovascular diseases. We and others had shown that young people lacked the knowledge regarding the risk factors associated with PCAD and had poor lifestyle practices, and they were less likely to discuss these issues with healthcare providers [19,20,21]. Consequently, it is imperative to investigate the prevalence of cardiovascular risk factors in Saudi Arabia’s younger population in order to prevent the incidence of morbidity and mortality of PCAD in the future. Thus, the present study aimed to evaluate the severity and identify the risk factors associated with PCAD in patients under the age of 50 at King Saud University Medical City (KSUMC), Saudi Arabia.

## 2. Methodology

This observational retrospective study was conducted between June 2022 and June 2023 at KSUMC, Riyadh, Saudi Arabia. We explored the association between coronary artery disease occlusion severity and types of ECG changes in PCAD patients who suffered a heart attack (coronary artery changes below 50 years of age).

The baseline and demographic characteristics of the study participants included age, gender, height, weight, and body mass index (BMI), while cardiovascular risk factors, such as type 2 diabetes mellitus (T2DM), hypertension, hyperlipidemia, smoking (current and previous), and family history, were also studied. Information about electrocardiogram (ECG), serum creatinine, hemoglobin A1C (HbA1C), total cholesterol, high-density lipoprotein (HDL), low-density lipoprotein (LDL), and triglycerides (TG) was also noted.

The inclusion criteria were Saudi patients with PCAD aged 18–50 years, confirmed by ECG findings and/or angiography (if available). The exclusion criteria were being non-Saudi, patients without PCAD, older than 50 or younger than 18, and patients with missing data. A total of 718 patients were included in the study after fulfilling the inclusion criteria. The patients were divided into three age groups: group 1 (<40 years), group 2 (40–45 years), and group 3 (>45–50 years). The study was approved by the Institutional Review Board of the College of Medicine, King Saud University (protocol # E-22-6747).

The Gensini score was calculated to assess the severity of occlusion of the coronary arteries. The Gensini score is a standard evaluation index that is widely used in similar studies. A score of zero indicates no occlusion. The Gensini score provides a reliable estimate of the severity of coronary involvement by assessing and grading the location and degree of vascular narrowing [9]. Additionally, the Gensini score is commonly used as a prognostic marker prior to coronary artery bypass grafting (CABG) procedures [10]. Out of 718 patients, the Gensini and vessel scores were calculated only for 283 patients who had angiographic reports in the integrated electronic system (e-SiHi) used at KSUMC. The angiographic reports were not available for all patients due to the implementation of the electronic system at the hospital in 2019. We collected data for patients from 2000 to 2022.

### Statistical Analysis

SPSS version 25 statistical software was used for data analysis. We obtained frequencies and percentages to describe the independent variables and the quantitative measures. Pearson’s Chi-square test was used to assess the association between the variables and age groups, and *p* ≤ 0.05 was considered statistically significant. In addition, ANOVA and post hoc analysis (HOC) tests were used for comparison among various study groups. Univariate regression analyses were performed to identify associations between risk factors and the age of premature coronary artery disease among the study participants.

## 3. Results

### 3.1. Sociodemographic and Clinical Characteristics of Patients (n = 718)

The sociodemographic and clinical characteristics of the 718 patients are described in Table 1. The mean age of patients in group 1 was 35.2 ± 4.5 (mean ± SD), in group 2 was 43.0 ± 1.3, and in group 3 was 48.4 ± 1.4. There were 540 male patients and 178 female patients in the study sample. Patients in group 2 (31.3 ± 10.5) had significantly higher BMI values compared to patients in group 3 (29.4 ± 5.3; *p* = 0.015). There was no significant association of HbA1C, total cholesterol, HDL, LDL, and TG (*p* > 0.05).

### 3.2. Comparison Between Age and Risk Factors (n = 718)

Correlations between age groups and risk factors (T2DM, hypertension, and smoking (current and past smokers)) using Chi-square analysis are described in Table 2. The number of patients with smoking habits in group 1 was significantly higher (97 (54.5%)) compared to patients in groups 2 and 3 (85 (47.8%) and 135 (37.3%), respectively; *p* = 0.001). A higher percentage of patients (9.7%) in the third group were ex-smokers. T2DM was significantly higher in group 3 patients (193 (53.3%)) compared to groups 1 and 2 patients (66 (37.1%) and 90 (50.6%), respectively; *p* = 0.001; Table 2). Hypertension was significantly higher in group 3 patients (189 (52.2%)) compared to groups 1 and 2 patients (59 (33.1%) and 76 (42.7%), respectively; *p* = 0.000; Table 2).

The levels of hyperlipidemia were not significant among the different age groups (*p* = 0.071). Family history-related factors, such as CAD, T2DM, hyperlipidemia, and hypertension, were not significant among different age groups (*p* = 0.091; Table 2). Although not statistically significant, a higher percentage of patients in groups 1 and 2 had a family history of hyperlipidemia compared to those in group 3.

Table 3 shows a univariate logistic regression model of risk factors. Obesity was significantly associated with PCAD (odds ratio = 1.4; *p* = 0.014), while hypertension had an odds ratio of 1.7 (*p* ≤ 0.001). In addition, patients with diabetes were 1.4 times more prone to PCAD in our sample size (*p* = 0.011), and those hyperlipidemia were 1.3 times (*p* = 0.073). Family history of CAD was also associated with PCAD, with an odds ratio of 1.0 (*p* = 0.046).

### 3.3. Comparison of Gensini Score with Age and Risk Factors (n = 283)

The comparison of the Gensini score with different age groups showed no significant association (*p* = 0.437; Table 4). The Gensini score > 39 in group 1 (age < 40 years) suggested a higher incidence of severe heart attacks (Table 4).

**Figure 1:**
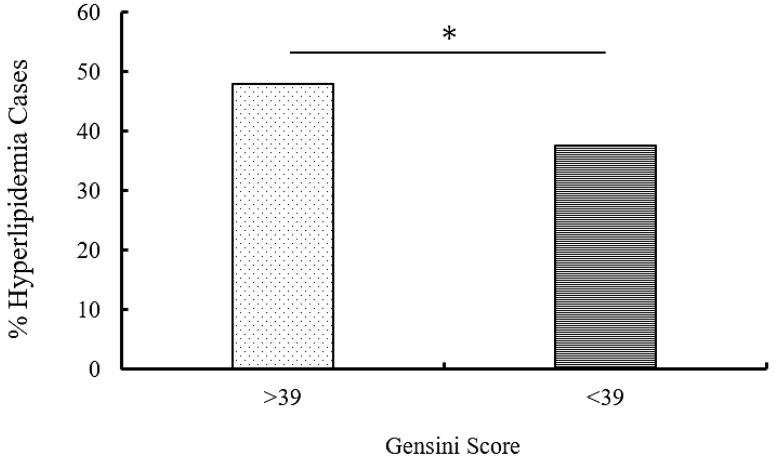
Comparison of Gensini scores with the hyperlipidemia risk factor. The percentage of patients with a Gensini score > 39 showed significantly higher lipidemic levels compared to patients with a Genisini score < 39; *p* = 0.05. *: Significant.

When the risk factors were compared with the Gensini score, hyperlipidemia levels were significantly higher in patients with a Gensini score > 39 compared to <39 [(47.9% (67) and 37.5% (54), respectively; *p* = 0.05; Table 5 and Figure 1)].

### 3.4. Comparison of Total Vessel Score and Age (n = 283)

Analysis of occlusion of specified arteries showed that approximately 55% of individuals in group 1 (<40 years of age) had two and three vessel occlusions (Table 6). There was no significant association of coronary lesions (single, two, and three vessel occlusions) among different age groups (*p* = 0.639; Table 6).

### 3.5. Comparison Between ECG Findings and Age (n = 718)

The number of patients with different ECG parameters (inferior ST elevation, non-specific ST-T changes, ST depression, and others (diffuse ST elevation and BBB)) was significantly higher in group 1 compared to groups 2 and 3 (*p* = 0.001; Table 7). Although non-significant, the number of patients with left main artery occlusion was higher in group 3 (8.6%) compared to groups 1 and 2 (4.6% and 4.5%, respectively; *p* = 0.229; Table 8).

## 4. Discussion

In this study, we investigated the prevalence of PCAD and its associated risk factors in Saudi patients below the age of 45 and between 45 and 50 years of age. The importance of our study is that PCAD is increasing at an early age, and information regarding PCAD is scarce in Saudi Arabia and globally. The pathogenesis of PCAD involves traditional (dyslipidemia, smoking, diabetes, obesity, and hypertension) and non-traditional risk factors (ethnicity and inflammation) [22]. In this study, we found significant differences in risk factors, like T2DM, hypertension, hyperlipidemia, and smoking, among different age groups.

We observed that almost 25% of our population suffered a heart attack below 40 years of age, with almost 51% of patients having more severe heart attacks with a higher Gensini score (>39). Interestingly, the smoking prevalence was about 54% in these patients, followed by T2DM (37%), hypertension (33%), and dyslipidemia (36%). This signifies the early onset of CAD/atherosclerosis with a constellation of modifiable risk factors.

Nearly 55% of the younger age group in the CAD population had two and three vessel occlusions. Although they are younger, the severity is concerning. This may be due to the high prevalence of risk factors in these groups, which can be controlled and modified. Smoking was significantly prevalent in the majority of patients under 40 years of age, while T2DM, hypertension, and hyperlipidemia were more common in patients in the 40–45 and 45–50 age groups.

There is an increased risk of PCAD associated with current smoking habits. In our study, a higher percentage of patients (9.7%) in the third group had a history of smoking. It is well known that smoking causes permanent damage to the arterial walls. Although the inflammation caused by smoke to the vessel wall decreases over time, arterial damage from long-term smoking exposes individuals to a major risk of CAD/PCAD, which is not reversible in a short period. There is a lack of data on the improvement of vascular endothelial function with smoking cessation. It may take a long time to achieve arterial wall stiffness similar to that of non-smokers [23,24].

Many studies have highlighted the importance of smoking as a major risk factor associated with PCAD [25,26,27]. An earlier study reported a significant prevalence of modifiable risk factors, such as hypertension, T2DM, and metabolic syndrome, in young smokers with PCAD compared to young smokers without PCAD [28]. A study by Sakr et al. found that individuals younger than 45 years of age with STEMI were predominantly smokers and had a high prevalence of dyslipidemia, while our study suggested the presence of these risk factors in individuals below 40 years of age [15]. Large-scale studies have reported an increased prevalence of smoking, hypertension, and family history in young patients with PCAD, consistent with our results [22,29]. A recent study conducted in Malaysia found that smoking, obesity, a family history of PCAD, total cholesterol levels, and a history of myocardial infarction were independent predictors of PCAD across all age groups [30].

In our study, patients in the older age group (group 3) had a higher percentage of family history of CAD compared to the younger groups (groups 1 and 2). This could be explained by the fact that younger patients may have parents of average age, in which vasculopathy could still be in its silent phase and yet to become symptomatic.

Morphological variations, either depression or elevation, in the ST segment have important clinical implications, such as myocardial ischemia and pericarditis [31]. In our study, the comparison of age with various angiographic findings revealed significantly higher-risk ECG features (inferior ST elevation) in younger patients (<40 years) compared to other groups (40–45 and 45–50 years of age). These findings highlighted that the younger patients were more prone to developing PCAD. The majority of patients with STEMI under 45 years of age were smokers, similar to our young cohort, or had high dyslipidemia but were unaware of their pre-existing condition. Several studies have shown that the percentage of atherosclerotic risk factors in young patients with acute myocardial infarction is higher than in older patients [32,33].

The Gensini scoring system is one of the most commonly used methods for the quantitative analysis of coronary artery disease. The Gensini score evaluates the number, location, and severity of coronary disease [9]. In our study, a comparison of the Gensini score with risk factors showed that hyperlipidemia was significantly correlated with increased severity of PCAD (47.9%), regardless of age. Other studies have also indicated that hyperlipidemia is an important predisposing factor for the incidence of PCAD [29,34,35]. In hyperlipidemia, low-density lipoproteins (LDLs) interact with the extracellular matrix through negatively charged proteoglycans and deposit within the vessel wall. These LDL deposits are then phagocytosed by macrophages via macrophage scavenger receptors and oxidized in lysosomes [36].

Macrophages containing oxidized lipids become foam cells due to their soapy appearance. These macrophages, with oxidized lipid content, become less mobile and secrete cytokines and inflammatory mediators, which lead to atherosclerotic lesions and plaque buildup, ultimately limiting blood flow to the heart muscle [37,38]. Therefore, hyperlipidemia is a significant risk factor for PCAD. Our recent study reported a high prevalence of unawareness regarding PCAD and unhealthy lifestyle practices in the young Saudi population, where individuals were unaware of their lipid profiles, consumed fast food, and lacked exercise [21]. These factors contribute to an increased lipid profile in the population, potentially hastening the onset of PCAD. Timely analysis of risk factors in the young population is crucial to reduce the morbidity and mortality associated with PCAD and other vascular diseases.

## 5. Conclusions

This study suggested that Saudi patients with PCAD under 40 years of age have a higher percentage of inferior ST elevation compared to older patients, while non-specific ST-T changes were significantly more common in older patients. Astonishingly, more than 50% of patients in all groups had two or three vessel occlusions. There was a high prevalence of modifiable risk factors, such as smoking, in younger patients, while hyperlipidemia was a risk factor for PCAD across all age groups. Additionally, hyperlipidemia was strongly correlated with severe vessel occlusion, as indicated by the Gensini score findings. Therefore, early preventive measures should be taken into consideration to reduce the future burden of cardiovascular complications in this population. To ensure broader applicability, the results should be replicated in other hospitals and centers across the country.

## Figures and Tables

**Table 1:** Sociodemographic and clinical characteristics of patients (*n* = 718).

Parameters	Total No. of Participants (*n* = 718)	Group 1, <40 Years, *n* = 178	Group 2, 40–45 Years, *n* = 178	Group 3, >45–50 Years, *n* = 362	*p*-Value
Age (years)	43.8 ± 5.9	35.2 ± 4.5 *^,^**	43.0 ± 1.3	48.4 ± 1.4 ***	0.000 *
Height (cm)	167.4 ± 8.3	169.2 ± 8.4 **	167.5 ± 9.3	166.5 ± 7.7	0.002 *
Weight (kg)	83.9 ± 17.5	85.9 ± 21.1 **	86.8 ± 18.1 ***	81.4 ± 14.8	0.001 *
BMI (kg/cm^2^)	30.0 ± 7.3	29.9 ± 6.9	31.3 ± 10.5 ***	29.4 ± 5.3	0.015 *
HbA1c (%)	7.5 ± 3.0	7.5 ± 4.5	7.4 ± 2.3	7.5 ± 2.3	0.926
TC (mmol/L)	4.5 ± 1.3	4.6 ± 1.7	4.5 ± 1.3	4.5 ± 1.2	0.639
HDL (mmol/L)	1.0 ± 0.3	1.0 ± 0.3	1.0 ± 0.3	1.0 ± 0.2	0.337
LDL (mmol/L)	2.7 ± 1.2	2.7 ± 1.4	2.6 ± 1.2	2.7 ± 1.1	0.742
TG (mmol/L)	1.8 ± 1.3	1.8 ± 1.6	1.9 ± 1.3	1.6 ± 0.9	0.141

Data are represented as mean and standard deviation (mean ± SD). ANOVA was used to evaluate significant differences between groups. * Below 40 is significant compared to 40–45, ** below 40 is significant compared to >45, and *** 40–45 is significant compared to >45. TG: triglycerides; LDL: low-density lipoprotein; HDL: high-density lipoprotein; TC: total cholesterol.

**Table 2:** Comparison of percentage distribution in different age groups for risk factors (*n* = 718).

Risk Factors	Group 1, <40 Years (*n* = 178)	Group 2, 40–45 Years (*n* = 178)	Group 3, >45–50 Years (*n* = 362)	*p*-Value
T2DM	66 (37.1%)	90 (50.6%)	193 (53.3%)	0.001 *
Hypertension	59 (33.1%)	76 (42.7%)	189 (52.2%)	0.000 *
Hyperlipidemia	64 (36.0%)	68 (38.2%)	158 (43.6%)	0.071
Smoking				0.001 *
Yes	97 (54.5%)	85 (47.8%)	135 (37.3%)	
Ex-smoker	14 (7.9%)	13 (7.3%)	35 (9.7%)	
Family history				0.091
None	140 (78.7%)	144 (80.9%)	256 (70.9%)	
CAD	32 (18.0%)	25 (14.0%)	91 (25.2%)	
T2DM	4 (2.2%)	7 (3.9%)	10 (2.8%)	
Hyperlipidemia	1 (0.56%)	1 (0.56%)	1 (0.27%)	
Hypertension	1 (0.56%)	1 (0.56%)	3 (0.82%)	

Data are presented as frequency and percentage. Significance was evaluated by the Chi-square test. T2DM: type 2 diabetes mellitus; CAD: coronary artery disease. *: Significant.

**Table 3:** Univariate logistic regression analysis of PCAD with risk factors (*n* = 718).

Variable	Level	OR	95% CI	*p*-Value
BMI (kg/m^2^)	Normal/ overweight (≤30) Obese (>30)	Ref. 1.447	1.077–1.945	0.014 *
T2DM	No Yes	Ref. 1.464	1.091–1.965	0.011 *
Hypertension	No Yes	Ref. 1.788	1.329–2.408	0.000 *
Hyperlipidemia	No Yes	Ref. 1.314	0.975–1.772	0.073
Smoking	Non-Smoker Smoker Non-Smoker	Ref. 0.572 1.008	0.330–0.991 0.584–1.740	0.001 *
Family history of CAD	No Yes	Ref. 1.064	0.173–6.566	0.046

The patients were divided into two groups: <40 years of age (*n* = 354) and 40 years and above (*n* = 362). OR: odds ratio; CI: confidence interval; BMI: body mass index; T2DM: type 2 diabetes mellitus. *: Significant.

**Table 4:** Comparison of percentage distribution in different age groups with low and high Gensini scores (*n* = 283).

Gensini Score	Group 1, <40 Years (*n* = 65)	Group 2, 40–45 Years (*n* = 66)	Group 3, >45–50 Years (*n* = 152)	*p*-Value
<39	31 (48.5%)	32 (48.5%)	80 (52.0%)	0.437
≥39	34 (51.5%)	34 (51.5%)	72 (48.0%)	

Data are represented as frequency and percentage. Significance was assessed by the Pearson Chi-square test. The median Gensini score was used to divide the groups.

**Table 5:** Comparison of risk factors with low and high Gensini scores (*n* = 283).

Parameters	Gensini Score < 39 (*n* = 143)	Gensini Score ≥ 39 (*n* = 140)	*p*-Value
T2DM	70 (48.6%)	78 (55.7%)	0.140
Hypertension	67 (46.5%)	64 (45.7%)	0.493
Hyperlipidemia	54 (37.5%)	67 (47.9%)	0.050 *
**Smoking**			0.409
Yes	77 (53.5%)	68 (48.6%)	
Ex-smoker	14 (9.7%)	16 (11.4%)	
**Family history**			0.546
None	92 (64.3%)	88 (62.9%)	
CAD	47 (32.9%)	49 (35.0%)	
T2DM	3 (2.1%)	2 (1.4%)	
Hyperlipidemia	0 (0.0%)	1 (0.7%)	
Hypertension	1 (0.7%)	0 (0.0%)	

Data are represented as frequency and percentage. Significance was assessed by the Pearson Chi-square test. The median Gensini score was used to divide the groups. T2DM: type 2 diabetes mellitus; CAD: coronary artery disease. *: Significant.

**Table 6:** Comparison of total vessel score and age (*n* = 283).

Coronary Artery Lesions	Group 1, <40 Years (*n* = 65)	Group 2, 40–45 Years (*n* = 66)	Group 3, >45–50 Years (*n* = 152)	*p*-Value
No occlusion	2 (3.1%)	1 (1.5%)	1 (0.7%)	0.639
Single vessel occlusion	27 (41.5%)	28 (42.4%)	61 (40.1)	
Two vessel occlusions	18 (27.7%)	17 (25.8%)	48 (31.6%)	
Three vessel occlusions	18 (27.7%)	20 (30.3%)	42 (27.6%)	

Data are presented as frequency and percentage. The Chi-square test was used to evaluate significant differences.

**Table 7:** Comparison of ECG findings with age (*n* = 718).

Parameters	<40 (*n* = 178)	40–45 (*n* = 178)	>45–50 (*n* = 362)	*p*-Value
Normal	45 (25.2%)	50 (28.0%)	81 (22.3%)	0.001 *
Anterior ST elevation (lateral and septal)	38 (21.3%)	20 (11.2%)	108 (29.8%)	
Inferior ST elevation	20 (11.2%)	18 (10.11%)	22 (6.0%)	
Non-specific ST-T changes	56 (31.4%)	57 (32.0%)	103 (28.4%)	
ST depression	6 (3.3%)	5 (2.8%)	11 (3.0%)	
Q-wave	7 (3.9%)	13 (7.3%)	11 (3.0%)	
Others (diffuse ST elevation and BBB)	6 (3.3%)	15 (8.4%)	26 (7.1%)	

*: Significant.

**Table 8:** Comparison of age with specified artery occlusion (*n* = 283).

Stenosed Vessels	Group 1, <40 Years	Group 2, 40–45 Years	Group 3, >45–50 Years	*p*-Value
Left anterior descending	55 (84.6%)	55 (83.3%)	130 (85.5%)	0.806
First diagonal	10 (15.4%)	12 (18.5%)	26 (17.1%)	0.818
Second diagonal	5 (7.7%)	4 (6.1%)	14 (9.2%)	0.609
Left main	3 (4.6%)	3 (4.5%)	13 (8.6%)	0.229
Right coronary	37 (56.9%)	42 (63.6%)	82 (53.9%)	0.575
Posterior descending	2 (3.1%)	6 (9.1%)	13 (8.6%)	0.210
Left circumflex	24 (36.9%)	24 (36.4%)	63 (41.4%)	0.470
Obtuse	9 (13.8%)	12 (18.2%)	36 (23.7%)	0.087
Posterolateral	2 (3.1%)	4 (6.1%)	4 (1.4%)	0.441
Apical	2 (3.1%)	3 (4.5%)	2 (1.3%)	0.316

Data are presented as frequency and percentage. Significance was evaluated by the Chi-square test.

## Data Availability

The complete data and materials used and analyzed in the current study are available from the corresponding author upon reasonable request.
